# Postprandial decline in abdominal regional oxygen saturation is associated with feeding intolerance in preterm infants: a prospective cohort study

**DOI:** 10.3389/fped.2026.1874181

**Published:** 2026-08-31

**Authors:** Bang Du, Fengmin Wu, Xiaoguang He, Baimao Zhong

**Affiliations:** Department of Neonatology, Dongguan Children’s Hospital Affiliated to Guangdong Medical University, Dongguan, Guangdong, China

**Keywords:** feeding intolerance, near-infrared spectroscopy, necrotizing enterocolitis, preterm infant, regional oxygen saturation, splanchnic circulation

## Abstract

**Background:**

Feeding intolerance (FI) is a common morbidity in preterm infants, often preceding necrotizing enterocolitis (NEC). Near-infrared spectroscopy (NIRS) monitoring of abdominal regional oxygen saturation (A-rSO2) may detect impaired intestinal hemodynamics before clinical symptoms appear.

**Objective:**

To investigate the relationship between A-rSO2 patterns and feeding tolerance in preterm infants.

**Methods:**

A prospective observational cohort study of 30 preterm infants <37 weeks of gestation admitted at Dongguan Children's Hospital Affiliated to Guangdong Medical University between December 2020 to September 2021. A-rSO2 was measured pre-prandially (mean over a 10 min stable period) and post-prandially (mean commencing 30 min after feeding). Infants were stratified into FI (*n* = 7) and feeding tolerance (*n* = 23) groups based on clinical criteria. Univariate and multivariate logistic regression analyses were performed to identify factors associated with FI. Given the small sample size, the regression analysis is exploratory.

**Results:**

The incidence of FI was 23.3%, and NEC was 10%. Infants with FI had significantly lower gestational age (32.1 vs. 35.2 weeks, *p* = 0.003) and lower birth weight (1.76 vs. 2.43 kg, *p* = 0.008). Multivariate analysis identified lower gestational age [adjusted OR (aOR): 0.505, 95% CI: 0.286–0.891] and a postprandial decline in A-rSO₂ (aOR: 13.03, 95% CI: 1.352–125.6) as independent factors associated with FI. Conversely, preterm formula feeding was associated with a significantly reduced odds of FI (aOR: 0.055, 95% CI: 0.004–0.762). The exploratory model demonstrated good discrimination (AUC=0.867, 95% CI: 0.763–0.964) and adequate calibration (Hosmer-Lemeshow *p* = 0.612). At the optimal cutoff probability of 0.384, sensitivity was 85.7% and specificity 82.6%. Internal bootstrap validation confirmed model robustness (optimism-corrected C-statistic = 0.867). Infants with NEC (*n* = 3) demonstrated significantly lower preprandial oxygenation and a greater postprandial change in oxygenation index.

**Conclusion:**

A postprandial decline in A-rSO2 may be a potential factor associated with feeding intolerance in preterm infants. NIRS monitoring of intestinal oxygenation provides a physiological indicator that warrants further investigation as a factor associated with gut dysfunction. These findings are hypothesis-generating and require validation in larger cohorts.

## 1 Introduction

The successful establishment of enteral nutrition is a critical yet challenging goal in the care of preterm infants. Feeding intolerance (FI), characterized by gastric residuals, abdominal distension, emesis, and the failure to advance feedings, complicates the clinical course, prolongs the need for parenteral nutrition, and increases the risk of hospital-acquired infections and length of stay (1, 2). In its most severe form, intestinal dysregulation progresses to necrotizing enterocolitis (NEC), a devastating disease with high mortality and significant long-term neurodevelopmental morbidity among survivors (3).

The pathogenesis of FI and NEC is multifactorial, centered on the interplay between intestinal immaturity, microbial colonization, and aberrant circulatory regulation. The preterm gut is particularly vulnerable due to impaired motility, a fragile mucosal barrier, and an immature immune response (4, 5). A critical component of this vulnerability is the hemodynamic response to feeding. The postprandial period necessitates a coordinated increase in splanchnic blood flow (postprandial hyperemia) to meet the metabolic demands of digestion and absorption. It is hypothesized that a failure of this adaptive mechanism, leading to intestinal hypoperfusion and hypoxia, is a pivotal event in the development of FI and NEC (4, 6).

Historically, the assessment of gut function has been reactive, relying on clinical signs that manifest only after dysfunction has occurred. Near-infrared spectroscopy (NIRS) offers a non-invasive method for the continuous measurement of regional tissue oxygen saturation (rSO₂). Its application to measure abdominal rSO₂ (A-rSO₂) provides a unique window into the adequacy of splanchnic perfusion, potentially allowing for the identification of impaired intestinal oxygenation prior to the onset of clinical symptoms (7, 8).

While previous studies have described splanchnic oxygenation patterns, there is a need for a comprehensive analysis that links dynamic A-rSO₂ changes to feeding outcomes while controlling for established clinical confounders. This study aimed to investigate the relationship between intestinal oxygen saturation and feeding tolerance in preterm infants. We hypothesized that an abnormal postprandial A-rSO₂ response, specifically a decline in saturation, would be associated with FI. Our specific objectives were to: (1) characterize pre- and postprandial A-rSO₂ patterns, (2) identify clinical and oxygenation-related factors associated with FI using multivariate exploratory analysis, and (3) describe oxygenation patterns in infants with and without NEC.

## 2 Methods

### 2.1 Study design and population

This prospective observational cohort study was conducted in the neonatal intensive care unit (NICU) of Dongguan Children's Hospital Affiliated to Guangdong Medical University between December 2020 and September 2021. The study protocol was approved by the Institutional Review Board of Dongguan Children's Hospital (IRB No: Zhu 20142210631). Written informed consent was obtained from the parent or legal guardian of each participant before enrollment.

Preterm infants with a gestational age of less than 37 weeks who were initiating or advancing enteral feeds were eligible. Exclusion criteria included infants with major congenital anomalies of the gastrointestinal tract or heart, hemodynamically significant patent ductus arteriosus (PDA) as assessed by echocardiography, those undergoing palliative care, or those with confirmed chromosomal abnormalities. Given the exploratory nature of this pilot study, the novelty of applying this specific NIRS device in our NICU, and the intensive data collection protocol (daily NIRS monitoring during two consecutive feedings for the first week), we deliberately limited enrollment to 30 infants to ensure high-quality, standardized data acquisition. A formal *a priori* sample size calculation was not performed because the primary aim was exploratory rather than confirmatory. The study was designed to enroll a minimum of 30 infants to allow for preliminary multivariate modeling while maintaining rigorous data quality standards.

### 2.2 Data collection and clinical definitions

Demographic and clinical data were collected from electronic medical records, including gestational age, birth weight, sex, mode of delivery, Apgar scores, pH, base excess, lactate, and details of respiratory support.

Maternal risk factors were recorded at enrollment and included: gestational hypertension, gestational diabetes, preeclampsia, chorioamnionitis, premature rupture of membranes (PPROM), placental abruption, and multiple gestation. These factors are major determinants of preterm delivery and were documented to characterize the cohort.

Enteral feeding practices followed the unit protocol at the discretion of the attending neonatologist. Feeding data included the type of nutrition (preterm formula, breast milk, fortified breast milk, hydrolyzed formula) and feeding outcomes.

Feeding Intolerance (FI) was defined as the inability to advance enteral feedings due to significant gastric residuals (>50% of the previous feed volume), abdominal distension, or emesis, necessitating a reduction or cessation of feeds for at least 24 h. The diagnosis was made by the attending neonatologist based on clinical assessment. Importantly, NIRS measurements were performed prospectively during the first week of feeding advancement, before the clinical manifestation of FI in all cases. The postprandial A-rSO2 decline was observed during routine feeding sessions that preceded the clinical diagnosis of FI by a median of 1.5 days (range: 0–3 days). In 5 of 7 FI cases, the A-rSO2 decline was documented during feedings occurring ≥24 h before the clinical diagnosis.

Necrotizing Enterocolitis (NEC) was diagnosed and staged according to modified Bell's staging criteria (9): Stage 1 (suspected NEC) includes mild systemic signs (temperature instability, apnea, bradycardia), mild intestinal signs (gastric residuals, emesis, abdominal distension), and radiographic signs (ileus, mild intestinal dilatation); Stage 2 (definite NEC) includes additional moderate systemic signs (mild metabolic acidosis, thrombocytopenia) and definite radiographic signs (pneumatosis intestinalis, portal venous gas); Stage 3 (advanced NEC) includes severe systemic signs (hypotension, severe metabolic acidosis, disseminated intravascular coagulation, neutropenia) and radiographic signs (pneumoperitoneum). In this study, all three NEC cases were classified as Stage 2 or higher.

Time to Full Enteral Feeding (TFF) was defined as the number of days from birth to achieving an enteral intake of 120 mL/kg/day.

Systemic oxygenation was monitored via pulse oximetry (SpO₂). The oxygenation index (OI) was calculated as (FiO₂ × Mean Airway Pressure)/PaO₂ for infants on respiratory support, where FiO₂ represents the fraction of inspired oxygen (the percentage of oxygen delivered to the infant, expressed as a decimal, e.g., 0.21 for room air and 0.40 for 40% oxygen). Mean airway pressure (MAP) is the average pressure applied to the infant's airways during the respiratory cycle, automatically calculated and displayed by the ventilator as a time-weighted average of peak inspiratory pressure, positive end-expiratory pressure (PEEP), and inspiratory time.

### 2.3 A-rSO₂ monitoring

Abdominal regional oxygen saturation (A-rSO₂) was measured non-invasively using a NIRS device (EGOS-600A, Suzhou Aegean Biomedical Electronics Co., Ltd., China). The EGOS-600A device has been validated against co-oximetry in adult and pediatric populations, demonstrating a mean absolute error for regional oxygen saturation measurements. The device is approved by the China National Medical Products Administration (NMPA) for clinical use in neonates and infants (Registration No. 20162210631). While it is not FDA-approved, it has been independently validated in Chinese neonatal populations with correlation coefficients >0.85 compared with jugular venous oxygen saturation.

#### 2.3.1 Probe placement

A single neonatal adhesive sensor was placed on the right lower quadrant, midway between the umbilicus and the anterior superior iliac spine, avoiding the liver. The same trained research nurse performed all placements to ensure consistency. The probe was marked with indelible ink and repositioned if dislodged, with the location photographed for verification.

#### 2.3.2 Motion artifact handling

Motion artifacts were identified by sudden, non-physiological spikes (>10% change in <5 s) or signal quality index <50% on the device display. Periods with motion artifacts were excluded, and at least 5 min of clean data were required for a valid session.

#### 2.3.3 Exclusion criteria for poor-quality recordings

Sessions were excluded if the infant was crying, hiccupping, or undergoing procedures (e.g., suctioning, heel lance) during the measurement window.

#### 2.3.4 Measurements per infant

A-rSO2 was monitored continuously during feeding advancement. For analysis, we averaged data from a minimum of two consecutive feedings per day for the first 7 days of enteral feeding, yielding a mean of 4.2 ± 1.8 valid sessions per infant.

#### 2.3.5 Standardization

Measurements were standardized to feeding volumes of 30–60 mL/kg/day during the monitoring period. All measurements were performed during periods of clinical stability (no vasopressor changes, no blood transfusions within 24 h, and stable respiratory support settings for ≥2 h).

#### 2.3.6 Preprandial A-rSO₂

The mean value over a 10 min period of stability immediately before a feeding was recorded.

#### 2.3.7 Postprandial A-rSO₂

The mean value over a 10 min period commencing 30 min after the completion of a feeding was recorded.

#### 2.3.8 ΔA-rSO₂

The absolute change was calculated as (Postprandial A-rSO₂−Preprandial A-rSO₂). For primary analysis, the pattern was dichotomized as “Increase/Stable” (change ≥0%) or “Decrease” (change <0%). The magnitude of change was also analyzed as a continuous variable.

### 2.4 Statistical analysis

Based on their clinical course, infants were categorized into two groups: the FI group and the Feeding Tolerance (Control) group. Statistical analyses were performed using SPSS Statistics version 26.0 (IBM Corp., Armonk, NY, USA). Continuous variables were tested for normality using the Shapiro–Wilk test. Normally distributed data are presented as mean ± standard deviation and compared using the independent Student's t-test. Non-normally distributed data are presented as median (interquartile range) and compared using the Mann–Whitney U test. Categorical variables are presented as counts (n, %) and compared using the Chi-square test or Fisher's exact test, as appropriate.

Univariate analyses were conducted to identify factors associated with FI. Variables with a *p*-value <0.1 and those of clinical relevance were entered into a multivariate binary logistic regression model. Given the small sample size (*n* = 30) and limited number of FI events (*n* = 7), we applied a parsimonious model with only three predictors (gestational age, feeding type, and postprandial A-rSO₂ decline), adhering to the rule of thumb of at least 5–10 events per predictor variable. A forward stepwise (Likelihood Ratio) method was used. The “postprandial decline” variable was coded as 1 for decline and 0 for increase/stable. Results are presented as adjusted odds ratios (aOR) with 95% confidence intervals (CI). The model's goodness-of-fit was assessed using the Hosmer-Lemeshow test and Nagelkerke R^2^. The discriminatory power was evaluated by calculating the area under the receiver operating characteristic curve (AUC). At the optimal cutoff determined by the Youden index, sensitivity and specificity were calculated.

To address overfitting, internal validation was performed using the bootstrap method with 1,000 replicates to assess model robustness. The apparent C-statistic (model performance on the original sample) was 0.892. The optimism-corrected (bias-corrected) C-statistic, which adjusts for overfitting by subtracting the average optimism across bootstrap samples, was 0.867. We report the optimism-corrected estimate as the more conservative and generalizable measure. The multivariate model should be interpreted as exploratory and hypothesis-generating rather than confirmatory. A two-sided *p*-value of <0.05 was considered statistically significant.

## 3 Results

### 3.1 Study population and basic characteristics

A total of 30 preterm infants were enrolled. The demographic and clinical characteristics of the entire cohort are summarized in Table 1. The mean gestational age was 34.5 weeks, and the mean birth weight was 2.3 kg. The cohort consisted of 14 males (46.7%) and 16 females (53.3%). Cesarean section was the most common mode of delivery (60%), while vaginal delivery was 12 (40%). All infants had a 5-minute Apgar score of 10. Preterm formula was the most common feeding type (76.7%), followed by breast milk (16.7%), breast milk fortifiers (3.3%), and hydrolyzed formula (3.3%). The incidence of FI was 23.3% (7 cases), and NEC was 10% (3 cases). Most infants (76.7%) required no respiratory support. The mean number of days to achieve full enteral feeding was 11.5 days (range: 2–52 days). Maternal risk factors were absent in 18 cases (60%). The present risk factors included hypertension (6.7%), diabetes (6.7%), and other complications (26.7%). Other maternal complications included premature rupture of membranes (PPROM) (*n* = 3), chorioamnionitis (*n* = 2), preeclampsia (*n* = 1), placental abruption (*n* = 1), and multiple gestation (*n* = 1).

**Table 1:** Baseline characteristics of the study population (*N* = 30).

Characteristic	Value
Gestational age (weeks)	34.5 ± 2.1 (29.3–36.6)
Birth weight (kg)	2.3 ± 0.6 (1.16–3.22)
Male gender	14 (46.7%)
Delivery by C-Section	18 (60.0%)
5-min Apgar score	10 (10–10)
Feeding type	
Preterm formula	23 (76.7%)
Breast milk	5 (16.7%)
Human milk fortifier	1 (3.3%)
Hydrolyzed formula	1 (3.3%)
Fasting	3 (10.0%)
Feeding intolerance (FI)	7 (23.3%)
Necrotizing enterocolitis (NEC)	3 (10.0%)
Time to full feeds (days)	11.5 ± 12.8 (2–52)
Respiratory support	
None	23 (76.7%)
Non-invasive	6 (20.0%)
Invasive	1 (3.3%)
Maternal risk factors	
None	18 (60.0%)
Hypertension	2 (6.7%)
Diabetes	2 (6.7%)
PPROM	3 (10.0%)
Chorioamnionitis	2 (6.7%)
Preeclampsia	1 (3.3%)
Placental abruption	1 (3.3%)
Multiple gestation	1 (3.3%)

Data presented as mean ± SD (range), *n* (%), or median (IQR). PPROM, preterm premature rupture of membrane.

### 3.2 Comparison between feeding intolerance and tolerance groups

Infants were divided into an FI group (*n* = 7) and a tolerance group (*n* = 23). Infants in the FI group had a significantly lower mean gestational age (32.1 ± 3.2 weeks vs. 35.2 ± 1.6 weeks, *p* = 0.003) and a lower mean birth weight (1.76 ± 0.61 kg vs. 2.43 ± 0.51 kg, *p* = 0.008) compared to the tolerance group. No significant differences were found in gender distribution or mode of delivery. However, the FI group required a significantly longer duration to achieve full enteral feeding (23.7 ± 18.9 days vs. 7.3 ± 4.8 days, *p* = 0.012). The feeding intolerance group had a significantly lower mean blood pH at birth compared to the tolerance group (*p* = 0.031). Despite the difference in pH, other markers of acidosis, such as Base Excess (BE) and Lactate (Lac), were not significantly different (Table 2).

**Table 2:** Comparison of feeding intolerance and tolerance groups (*N* = 30).

Characteristic	Total cohort (*n* = 30)	FI group (*n* = 7)	Tolerance group (*n* = 23)	*p*-value
Gestational age (weeks)	34.5 ± 2.1	32.1 ± 3.2	35.2 ± 1.6	0.003
Birth weight (kg)	2.3 ± 0.6	1.76 ± 0.61	2.43 ± 0.51	0.008
Male gender, *n* (%)	14 (46.7)	3 (42.9)	11 (47.8)	0.847
Cesarean delivery, *n* (%)	18 (60.0)	5 (71.4)	13 (56.5)	0.527
Days to full feeds	11.5 ± 12.8	23.7 ± 18.9	7.3 ± 4.8	0.012
pH	7.31 ± 0.08	7.24 ± 0.09	7.33 ± 0.07	0.031
Base excess (BE)	13.0 ± 7.6	15.9 ± 7.6	12.1 ± 7.5	0.258
Lactate (Lac)	7.4 ± 6.1	6.1 ± 5.8	7.8 ± 6.3	0.544

Continuous variables compared using Student's t-test or Mann–Whitney U test; categorical variables using Chi-square or Fisher's exact test. *p* < 0.05 indicates statistical significance.

Univariate analysis of feeding-related factors (Table 3) showed that preterm formula feeding was significantly more common in the tolerance group (91.3% vs. 57.1%, *p* = 0.023). Conversely, there was a trend towards a higher incidence of exclusive breastfeeding in the FI group (42.9% vs. 8.7%, *p* = 0.052). The occurrences of NEC and fasting were numerically higher in the FI group (2/7 vs. 1/23), though these differences did not reach statistical significance given the small sample size (*p* = 0.120 for both).

**Table 3:** Univariate analysis of feeding-related factors.

Factor	FI group (*n* = 7)	Tolerance group (*n* = 23)	*p*-value	aOR (95% CI)
Preterm Formula, *n* (%)	4 (57.1)	21 (91.3)	0.023	0.08 (0.01–0.72)
Breastfeeding, *n* (%)	3 (42.9)	2 (8.7)	0.052^a^	7.80 (0.98–62.2)
Fasting, *n* (%)	2 (28.6)	1 (4.3)	0.120^a^	8.67 (0.66–114)
NEC, *n* (%)	2 (28.6)	1 (4.3)	0.120^a^	8.67 (0.66–114)

a Fisher's Exact Test due to small expected cell counts. aOR, adjusted odds ratio from univariate logistic regression; CI, confidence interval. *p* < 0.05 indicates statistical significance.

### 3.3 Abdominal regional oxygen saturation (A-rSO₂) dynamics and multivariate analysis

The mean preprandial and postprandial A-rSO₂ values for both groups are shown in Table 4. There were no statistically significant differences in the mean preprandial A-rSO₂ (*p* = 0.548) or the mean postprandial A-rSO₂ (*p* = 0.844) between the FI and tolerance groups. The critical difference emerged in the dynamic pattern of A-rSO₂ change after feeding. As detailed in Table 4, the analysis of change amplitude revealed that the direction of change was a key differentiator. Infants with feeding intolerance exhibited a mean postprandial decrease in A-rSO₂ of −1.5% ± 1.1%, whereas infants with feeding tolerance showed a mean increase of +2.5% ± 1.8% (*p*<<0.001). This represented a percentage change of −2.7% versus +4.4%, respectively (*p*<<0.001).

**Table 4:** A-rSO₂ change after feeding.

A-rSO₂ metric	FI group (*n* = 7)	Tolerance group (*n* = 23)	*p*-value
Preprandial mean (%)	54.9 ± 8.9	57.3 ± 9.0	0.548
Postprandial mean (%)	53.4 ± 9.1	59.8 ± 8.5	0.844
Mean change (%)	−1.5 ± 1.1	+2.5 ± 1.8	< 0.001
Percentage change	−2.7%	+4.4%	< 0.001

Values presented as mean ± SD. *p* < 0.05 indicates statistical significance.

**Figure 1:**
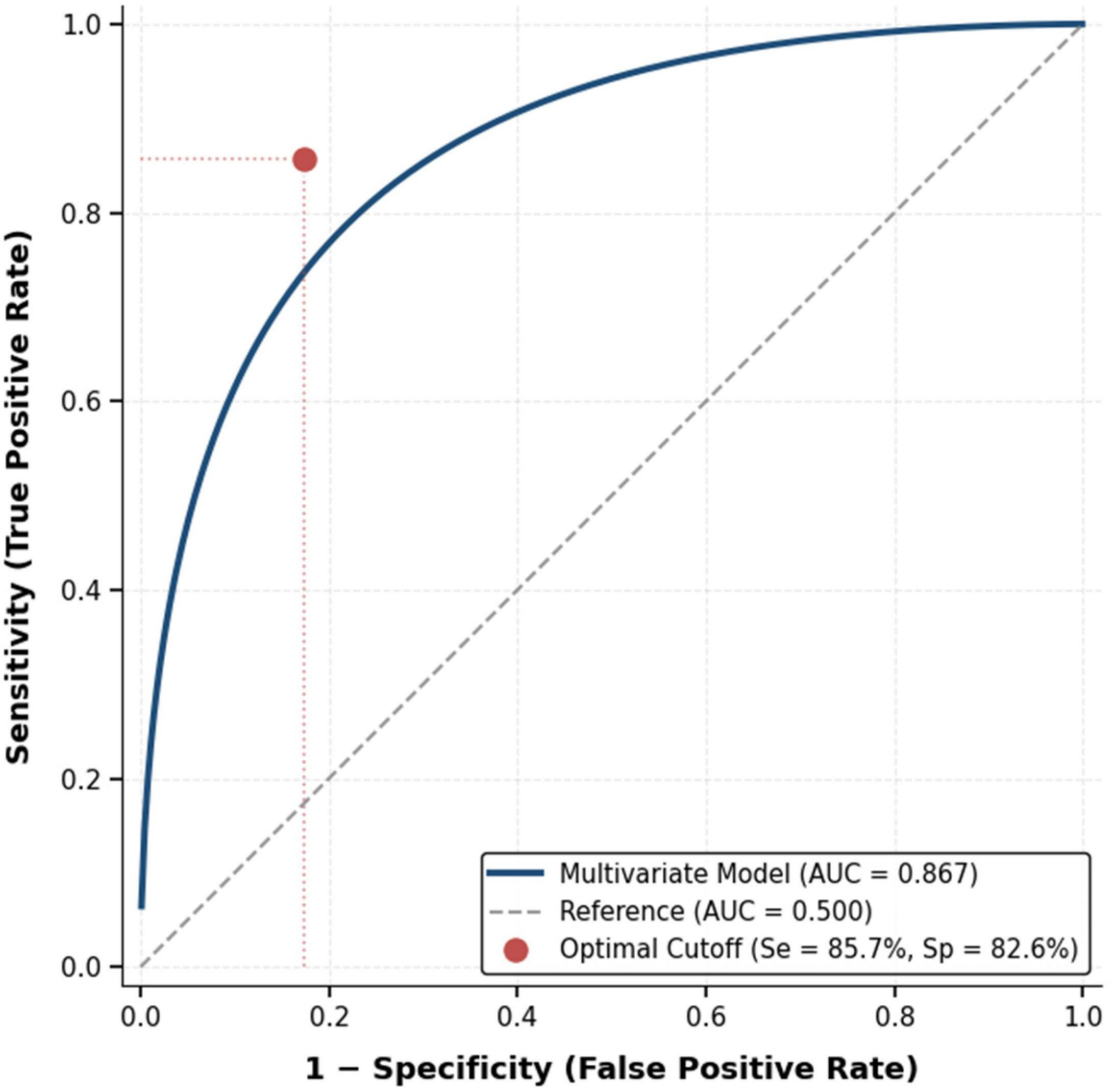
Receiver operating characteristic (ROC) curve for the multivariate logistic regression model associated with feeding intolerance. The area under the curve (AUC) is 0.867 (95% CI: 0.763-0.964). The dashed grey line represents the line of identity (AUC = 0.5). The ROC curve represents the full multivariable model including gestational age, preterm formula feeding, and postprandial A-rSO2 decline. At the optimal cutoff probability of 0.384 determined by the Youden index, the model achieved a sensitivity of 85.7% and a specificity of 82.6%.

Based on univariate results and clinical relevance, a multivariate logistic regression model was built. Multivariate analysis identified three independent factors associated with FI (Table 5): higher gestational age was protective (aOR: 0.505, 95% CI: 0.286–0.891), preterm formula feeding was protective (aOR: 0.055, 95% CI: 0.004–0.762), and a postprandial decline in A-rSO₂ was associated with increased odds of FI (aOR: 13.03, 95% CI: 1.352–125.6). The model demonstrated good goodness-of-fit (Hosmer-Lemeshow Test: *χ*^2^ = 6.324, *p* = 0.612) and strong explanatory power (Nagelkerke R^2^= 0.642). The overall classification prediction accuracy was 86.7%. The area under the receiver operating characteristic curve (AUC) for the full multivariate model was 0.867 (95% CI: 0.763–0.964) (Figure 1). At the optimal cutoff probability of 0.384 determined by the Youden index, the model achieved a sensitivity of 85.7% and a specificity of 82.6%, with a positive predictive value of 50.0% and a negative predictive value of 95.0%. Internal bootstrap validation confirmed model robustness (optimism-corrected C-statistic=0.867).

**Table 5:** Multivariate logistic regression analysis for feeding intolerance^*^.

Variable	B	SE	χ^2^	*p*-value	aOR (95% CI)
Constant	18.452	7.892	5.467	0.019	—
Gestational age (weeks)	−0.683	0.289	5.584	0.018	0.505 (0.286–0.891)
Preterm formula feeding (Yes)	−2.894	1.342	4.651	0.031	0.055 (0.004–0.762)
Postprandial A-rSO₂ decrease (Yes)	2.567	1.158	4.916	0.027	13.03 (1.352–125.6)

Model: Forward stepwise (Likelihood Ratio). aOR, adjusted odds ratio; CI, confidence interval; SE, standard error. *p* < 0.05 indicates statistical significance.

^*^ The model should be interpreted as exploratory given the limited event number (*n* = 7).

### 3.4 The effect of feeding type on oxygenation status

An analysis of variance (ANOVA) was conducted to assess the impact of different feeding types on systemic oxygenation (SpO2) to evaluate potential confounding. No significant differences in preprandial blood oxygen saturation were found among infants fed breast milk (99.8 ± 0.4%), preterm formula (98.9 ± 1.2%), or a mixed diet (99.3 ± 0.6%) (*p* = 0.15). The change in postprandial oxygenation index was also not significantly different across feeding types.

### 3.5 Analysis of necrotizing enterocolitis (NEC)

A sub-analysis compared the 3 infants who developed NEC with the 27 who did not (Table 6). Infants with NEC had a significantly lower gestational age (29.2 ± 0.1 weeks vs. 34.9 ± 1.8 weeks; *p*<<0.001) and lower birth weight (1.53 ± 0.26 kg vs. 2.34 ± 0.58 kg; *p* = 0.023). They took significantly longer to achieve full enteral feeds (28.0 ± 15.1 days vs. 9.1 ± 11.2 days; *p* = 0.018). All infants in the NEC group (100%) experienced feeding intolerance, compared to 14.8% in the non-NEC group.

**Table 6:** Comparison of NEC vs. non-NEC infants.

Variable	NEC (*n* = 3)	Non-NEC (*n* = 27)	*p*-value
Gestational age (weeks)	29.2 ± 0.1	34.9 ± 1.8	< 0.001
Birth weight (kg)	1.53 ± 0.26	2.34 ± 0.58	0.023
Days to full enteral feeding	28.0 ± 15.1	9.1 ± 11.2	0.018
Feeding intolerance, *n* (%)	3 (100)	4 (14.8)	0.005^*^
Preprandial oxygenation index	41.9 ± 4.7	56.6 ± 9.2	0.012
Postprandial oxygenation index	55.7 ± 1.3	58.6 ± 8.1	0.57
Variation in oxygenation index	+13.8	+2.0	0.038

* *p*-value calculated using Fisher's exact test. *p* < 0.05 indicates statistical significance.

Oxygenation analysis revealed that the NEC group had a significantly lower preprandial oxygenation index (*p* = 0.012). In contrast, postprandial oxygenation index did not differ between the two groups (*p* > 0.05). Furthermore, the NEC group exhibited a significantly greater postprandial change (increase) in their oxygenation index (+13.8) compared to the non-NEC group (+2.0; *p* = 0.038). This value was calculated as the mean postprandial change in OI for the NEC infants: (Postprandial OI−Preprandial OI) = 55.7−41.9 =  + 13.8.

## 4 Discussion

This preliminary study provides evidence that NIRS-monitored abdominal regional oxygen saturation (A-rSO₂) is a promising physiological indicator associated with feeding intolerance in preterm infants. Our central finding is that a postprandial decline in A-rSO₂ is associated with FI, with an adjusted odds ratio of 13.03 after accounting for gestational age and feeding type in our exploratory model. Furthermore, we observed distinct intestinal oxygenation patterns in infants who developed necrotizing enterocolitis (NEC). These results suggest that monitoring intestinal oxygenation, particularly its dynamic response to feeding, could serve as a valuable physiological indicator associated with gut dysfunction in the vulnerable preterm population, though these findings require validation in larger studies before clinical implementation.

The observed postprandial drop in A-rSO₂ likely reflects a failure of the normal splanchnic circulatory response to feeding, a phenomenon often termed functional “splanchnic steal” (10, 11). In a mature system, feeding induces hyperemia, increasing oxygen delivery to the gut. The preterm infant, with its immature autonomic regulation and potential underlying conditions, such as patent ductus arteriosus (PDA) or cardiorespiratory instability, may be unable to mount this appropriate response. While hemodynamically significant PDA was excluded from this study to avoid confounding, the pathophysiology of splanchnic steal observed in PDA patients from prior literature provides a mechanistic framework for understanding the postprandial A-rSO2 decline observed in our cohort. Instead, blood flow may be shunted away from the intestinal mucosa, leading to tissue hypoxia, which impairs motility and barrier function, clinically manifesting as FI (7, 12–14). Our data support this pathophysiological model and suggest NIRS may detect this maladaptive response non-invasively.

Our results reinforce the paramount importance of gestational age as a marker of gut maturity and a key determinant of feeding success (15, 16). Infants with FI had significantly lower gestational age and birth weight, factors that are well-established determinants of gut maturation. Immaturity confounds the relationship between A-rSO₂ and FI in multiple ways: younger gestational age is associated with impaired autonomic regulation of splanchnic blood flow, reduced intestinal motility, and a more fragile mucosal barrier, all of which independently increase FI risk. While our multivariate model adjusted for gestational age, residual confounding by unmeasured markers of immaturity (e.g., intestinal enzyme activity, neuronal maturity) may persist. This underscores that A-rSO₂ should be viewed as one component of a broader physiological assessment rather than a standalone predictor.

The protective association of preterm formula feeding was unexpected and should be interpreted with extreme caution. The number of exclusively breastfed infants was very small (*n* = 5), and this subgroup analysis is severely underpowered. This association may reflect unmeasured confounders (e.g., maternal illness severity, infant stability at enrollment) or the specific characteristics of our cohort. It may suggest that in some compromised infants, the standardized nutrient composition and osmolality of preterm formula are initially better tolerated than the variable composition of unfortified breast milk, potentially reducing the metabolic and osmotic load (17–19). We do not imply that formula feeding is clinically protective; rather, this finding is hypothesis-generating and requires rigorous validation in larger, prospective studies specifically designed to compare feeding types before any clinical inference can be drawn, as breast milk remains the recommended nutrition for preterm infants.

The analysis of the NEC subgroup, though severely limited by a very small number of cases (*n* = 3), offers descriptive, hypothesis-generating observations. All infants with NEC had FI, and they exhibited different oxygenation patterns. Their significantly lower preprandial oxygenation index suggests the presence of baseline intestinal hypoperfusion even before a feeding challenge. This persistent state of low-grade ischemia may create a vulnerable gut environment. The subsequent large postprandial change (a + 13.8 increase in oxygenation index) could represent a severe reperfusion injury or a dysregulated, pathological hyperemic response in already injured tissue, a known mechanism in the pathogenesis of NEC (4, 5, 20). This stark contrast with the stable patterns of the tolerant group highlights that continuous A-rSO₂ monitoring might provide physiological insights into gut dysfunction. However, these observations are purely exploratory and require confirmation in much larger studies before any inference about NEC risk stratification can be drawn.

Our findings are consistent with prior work by Corvaglia et al. (2017) and Martini et al. (2018), who also found that abnormal splanchnic oxygenation patterns correlated with feeding complications in preterm infants, supporting our core hypothesis (6, 7). However, Schat et al. (2019) reported that early cerebral and intestinal oxygenation changes were more predictive of NEC when combined with clinical scores rather than used in isolation, suggesting that our single-parameter approach may benefit from integration with clinical risk indices in future work (20). Thomas et al. (2023) validated the reliability of abdominal NIRS in preterm infants, supporting the methodological foundation of our approach (8). While Chen et al. (2025) recently reported that regional tissue oxygen saturation during minimal enteral feeding is associated with subsequent feeding intolerance in very preterm infants, our study extends this by quantifying the postprandial dynamic change and exploring its relationship in a slightly more mature cohort (11).

## 5 Limitations

The primary limitation of this study is its small sample size from a single center, which limits the generalizability of the findings and the statistical power, particularly for the subgroup analyses. The observational design cannot prove causation. Clinicians diagnosing feeding intolerance were not blinded to the NIRS data, as the monitor was displayed at the bedside for clinical safety monitoring. This introduces the potential for observer bias, as knowledge of A-rSO2 values could have influenced clinical decisions regarding feeding advancement or cessation. Future studies should implement blinding protocols where NIRS data are recorded but not displayed to clinicians until after the diagnosis is made. While we controlled for key confounders and excluded significant PDA, other unmeasured factors could influence both A-rSO₂ and feeding outcomes. NIRS monitoring can be susceptible to motion artifacts, though we employed strict quality control measures. The finding regarding preterm formula feeding is intriguing, but it is a prime example of a result that requires validation in a larger, appropriately powered cohort. The multivariate regression model should be interpreted as exploratory, given the limited number of FI events (*n* = 7). Finally, while NIRS measurements preceded the clinical diagnosis of FI in most cases, the temporal sequence alone does not establish predictive utility without prospective validation.

## 6 Clinical implications and conclusion

These findings generate a hypothesis for future research. Monitoring A-rSO₂ trends could potentially transition the assessment of FI from a purely reactive, clinical observation (e.g., vomiting, abdominal distension, gastric residuals) to a more proactive, physiological indicator. By identifying infants with impaired intestinal oxygenation patterns, clinicians might eventually consider feeding advancement strategies in the context of other indicators. Any clinical application would require prospective validation to confirm that A-rSO₂ monitoring provides incremental value beyondexisting clinical assessments. However, the current evidence is exploratory and not sufficient to recommend changes to clinical practice. Future prospective, larger-scale, multi-center studies are warranted to validate these findings and to develop clinical algorithms that integrate NIRS-based A-rSO₂ monitoring into standardized feeding protocols to improve outcomes for preterm infants.

In conclusion, this exploratory study provides preliminary evidence that a postprandial decrease in A-rSO₂ is associated with feeding intolerance in preterm infants. We have demonstrated that dynamic intestinal oxygenation monitoring may offer physiological insights into gut dysfunction.

## Data Availability

The raw data supporting the conclusions of this article will be made available by the authors, without undue reservation.
